# HIV counsellors’ knowledge and attitudes on HIV self-testing: A qualitative study in Eswatini

**DOI:** 10.4102/hsag.v30i0.2986

**Published:** 2025-07-31

**Authors:** Celiwe Z. Dlamini, Geertien C. Boersema, Gisela H. van Rensburg

**Affiliations:** 1 Department of Health Studies, College of Human Sciences, University of South Africa, Pretoria, South Africa

**Keywords:** attitudes, community-health services, Eswatini, HIV, HIV self-testing, HIV counsellors, knowledge and attitudes, targeted HIV self-testing

## Abstract

**Background:**

Human Immunodeficiency Virus (HIV) self-testing (HIVST) expands access to HIV diagnosis and holds potential for reaching high-risk and hard-to-reach populations. In Eswatini, HIV counsellors play a key role in providing HIVST, yet their knowledge and attitudes towards HIVST remain underexplored.

**Aim:**

This study aimed to explore the knowledge and attitudes of HIV counsellors in offering HIVST as a strategy to enhance targeted HIV services in Eswatini.

**Setting:**

The study was conducted at the outpatient department of a regional hospital in Manzini, Eswatini.

**Methods:**

A qualitative, exploratory-descriptive research design was used. HIV counsellors were sampled using all-inclusive sampling. Individual, face-to-face semi-structured interviews were conducted with 13 HIV counsellors. The interviews were audio-recorded and transcribed. Data were thematically analysed.

**Results:**

HIV counsellors demonstrated a comprehensive understanding of their roles and responsibilities in delivering HIVST and expressed generally positive attitudes towards this testing approach. Recommendations were made for improving HIVST.

**Conclusion:**

The findings highlight the need for policymakers to prioritise the continuous professional development of HIV counsellors and equip them with strategies including mobile testing units and community-based HIVST distribution to reach marginalised high-risk populations within their specific area. Service delivery should integrate effective client feedback systems to improve HIVST, a continuously available support contact line, and government commitment to ensure consistent HIVST kit supplies to prevent disruptions in service delivery.

**Contribution:**

This study describes HIV counsellors in Eswatini’s knowledge and attitudes towards HIVST in Eswatini.

## Introduction

There is a global ambition to end AIDS and achieve the 2030 Sustainable Development Goals (SDGs), namely to end the public threat of Human Immunodeficiency Virus (HIV) and Acquired Immunodeficiency Syndrome (AIDS) (Frescura et al. 2022). Globally, strides have been made towards achieving the 95-95-95 targets of the Joint United Nations Programme on HIV/AIDS (UNAIDS 2023). Eswatini, Lesotho, Malawi, Zambia, and Zimbabwe have demonstrated commendable progress towards the HIV epidemic control (Centers of Disease Prevention and Control [CDC] 2024; World Health Organization [WHO] 2024). In Eswatini, the number of new HIV infections decreased from 14 000 in 2010 to 4800 in 2020 (WHO 2024), but Eswatini is still one of the countries with the highest HIV prevalence in the world (Nkambule et al. 2021). Disparities in treatment coverage still exist between geographic areas and subpopulations (Frescura et al. 2022). Certain subpopulation groups lag in awareness of their HIV status (UNAIDS 2020). The latest Eswatini population-based HIV impact assessment of 2021 highlighted the persistent risk of HIV acquisition among the HIV-negative population, including vulnerable groups such as adolescent girls, young women, and key populations including female sex workers, men having sex with men, people who inject drugs, and transgender people (Jones et al. 2021; Ministry of Health Eswatini [MoHE] 2021). The existence of high-risk, hard-to-reach populations with unknown HIV status necessitates the exploration of innovative strategies for HIV testing services, given the significant threat they pose to the attainment and sustainability of global and national HIV control efforts.

HIV self-testing (HIVST) is an acceptable and feasible strategy to reach the high-risk key populations (Boisvert Moreau et al. 2022). Studies demonstrated a high acceptability of HIVST among targeted populations, such as men having sex with men (Harichund & Moshabela 2018; Moradi et al. 2022) and female sex workers (Boisvert Moroeau et al. 2022). Even those with limited prior awareness of HIVST, including boyfriends of female sex workers, have shown interest in self-testing, affirming its acceptability (Boisvert Moreau et al. 2022). HIV self-testing can be used in diverse settings, including pharmacy settings (Mugo et al. 2017), further enhancing its feasibility. The options of performing the test onsite, for example at the clinic or at home, with or without clinicians’ guidance, extends the reach compared to previous tests such as the rapid test that required trained personnel.

HIV counsellors play a pivotal role in the delivery of HIV testing services, including HIVST services, acting as a primary interface between individuals seeking testing at primary health care (PHC) facilities and subsequent care. HIV counsellors need to provide the service as efficiently as possible to reach the targeted populations. The study aimed to explore and describe the knowledge and attitudes of HIV counsellors in offering HIVST as a strategy to enhance targeted HIV services in Eswatini. Understanding the perspectives, knowledge, and attitudes of these frontline healthcare professionals is paramount for the successful implementation of targeted HIVST interventions. By gaining insights into the challenges and strengths of HIV counsellors in the context of HIVST, this study contributes to the enhancement of HIV services.

## Research methods and design

### Research design

A qualitative, exploratory-descriptive research design was used in this study. This design was used to gain an in-depth understanding of HIV counsellors’ knowledge and attitudes towards HIVST.

### Study setting

The study was conducted at a regional referral hospital in the Manzini region of Eswatini. This hospital was situated in an industrial hub of the country. The facility’s outpatient department (OPD) at the hospital plays an important role in the provision of HIV services, catering to an average of 500 outpatients daily. The OPD also acts as a triage point before clients can be referred for specialised healthcare services within the hospital as well as to the national referral hospital for further management. Data were collected from the HIV counsellors working at this OPD.

### Study population and sampling strategy

The accessible population for the study were 20 HIV counsellors between the ages of 18 years and 60 years, providing HIVST at the hospital’s OPD. Census sampling was used because of the small population size (Brink, Van der Walt & Van Rensburg 2018), allowing equal opportunity for all the HIV counsellors to participate in the study. HIV counsellors received certification with the Eswatini Ministry of Health on HIV testing services, and they worked as an HIV counsellor for more than 12 months. Thirteen participants agreed to take part in the study.

### Data collection

Data were collected by the researcher through face-to-face interviews that lasted 40 min to 60 min each, using a semi-structured interview guide. A total of 13 interviews were conducted over 10 days between June and November 2022. Signed consent forms were completed following an explanation of the study’s purpose, potential benefits, expectations, and rights of the participants before the commencement of the interview. Interviews were conducted in the OPD’s counselling room after permission was received. This room was conducive because it was well-ventilated and private.

Participants’ demographic information, including their age, gender, highest educational qualification, HIV services training (including training on HIVST), and years of service as an HIV counsellor was collected with a separate self-report questionnaire just before the interview.

The interview guide consisted of four main questions with the first three questions aimed at exploring HIV counsellors’ knowledge and attitudes on HIVST. The fourth question was designed to allow the HIV counsellor to make recommendations and clarify support areas for HIV counsellors in the provision of HIVST. Field notes were taken regarding any technical issues experienced during the interview and important aspects that emerged during the interview such as patterns in the data or non-verbal cues. SiSwati, a local language, was used by participants during the interview only when they struggled to express themselves in English or by the researcher when participants could not understand the question.

### Data analysis

All demographic data responses were sequentially numbered and aligned with the recorded interviews and field notes. Audio-recordings were transcribed verbatim by a professional transcriber and any SiSwati data were translated during the transcriptions.

The interview transcripts were analysed inductively using thematic analysis. Braun and Clarke’s six-phased coding framework (Braun & Clarke 2022; Maguire & Delahunt 2017) was used to guide the coding and thematic analysis process, including familiarisation with the data, generating codes, searching for themes, reviewing themes, defining themes and writing up the report. Data were independently co-coded by an expert in qualitative data coding. Independent co-coding was followed by a consensus discussion to achieve clarity and depth in interpretation.

### Ethical considerations

An application for full ethics approval was made to the College of Human Sciences Research Ethics Review Committee of the University of South Africa, and approval was received on 29 November 2021 (reference no: 46609571_CREC_CHS_2021). The Scientific Ethics Committee of the Eswatini Ministry of Health provided approval for the study (EHHRRB152/2022). The researcher ensured that the research was conducted in an ethical manner as outlined in the Belmont Report (Polit & Beck 2018) adhering to the principles of beneficence, non-maleficence, respect for persons and justice. Participation in this study was voluntary, and participants signed written informed consent after the study purpose and objectives were explained.

## Results

The participants’ characteristics are described followed by the thematic data.

### Participants’ characteristics

Thirteen participants took part in the study and their ages ranged between 25 years and 40 years. The participants’ characteristics are outlined in Table 1.

**TABLE 1:** Participants’ characteristics.

Participant code	Age (years)	Gender	Years of experience in providing HIVST	Year of obtaining HIV testing services training
P01	30	Male	4	2017
P02	25	Female	4	2016
P03	31	Female	4	2016
P04	31	Female	4	2016
P05	35	Male	5	2012
P06	32	Female	4	2016
P07	31	Female	2	2013
P08	40	Female	4	2008
P09	32	Female	4	2014
P10	31	Male	4	2016
P11	31	Female	3	2017
P12	34	Female	4	2015
P13	35	Male	4	2016

HIVST, HIV self-testing.

Nine participants were female and four were males. This majority could be expected because male enrolment into the HIV testing services training is lower than females’ enrolment (Budu et al. 2019; McLaughlin, Muldoon & Moutray 2010). The experience with the provision of HIVST ranged between 2 and 5 years with the most having 4 years’ experience. All participants received their initial HIV Testing Services (HTS) training between 2008 and 2017. However, continuous annual refresher trainings are provided for HIV counsellors for continued capacity building.

### Thematic data

Three themes and nine subthemes were developed and are outlined in Table 2. These themes relate to the knowledge, attitudes and recommendations to improve HIVST provision as a targeted HIV service.

**TABLE 2:** Themes and subthemes.

Themes	Sub-themes
1. HIV counsellors’ knowledge enabling them to conduct HIVST as a targeted strategy	1.1 Knowledge of HIV counsellors’ roles and responsibilities 1.2 Knowledge to optimise targeting
2. Views on HIVST	2.1 Effectiveness of HIVST as a targeted strategy 2.2 Target population’s acceptability of HIVST 2.3 Accessibility to HIV services 2.4 Reduction of HIV counsellors’ workload
3. Recommendations for improving HIVST provision as a targeted HIV service	3.1 Client involvement 3.2 Building HIV counsellors’ HIVST capacity 3.3 Government support

HIVST, HIV self-testing; HIV, Human immunodeficiency virus.

#### Theme 1: HIV counsellors’ knowledge enabling them to conduct HIV self-testing as a targeted strategy

Participants showed knowledge of their roles and responsibilities in HIVST as part of the daily tasks, including screening for HIVST eligibility, educating clients on how to perform HIV self-tests, confirming positive HIV self-test screening results and offering linkages to treatment and HIV prevention services:

‘My key duties as an HIV counsellor are to ensure proper screening of clients and to ensure that HIV self-test is given to the high risk and unreachable population.’ (P03)

‘If a client was screened HIV positive using an HIV self-test, I confirm the result using an HIV rapid test as guided by the national HIV testing algorithm, if the results remain positive, I refer and link the clients to HIV care and treatment.’ (P02)

Knowledge of their roles and responsibilities enabled the HIV counsellors to provide the HIVST as a targeted strategy. HIV counsellors had unique insights to optimise the targeting of HIVST including knowledge of high-risk key populations and the ideal times to reach the targeted population with HIVST services. Participants described challenges to accessing HIVST services, for example, the stigma associated with LGBTQIA+ and limited time to test:

‘The high risk and unreachable population are the following, although not limited to: adolescent girls and young women, men of ages 29 to 39, drug injection users, female sex workers, sero-discordant couples and men having sex with men. So, most of the time they are in a hurry, they don’t have enough time to test, they fear stigma, and discrimination. So, with HIVST, I am able to maximize my HIV testing coverage of these populations by eliminating the barriers that these populations have to access HIV testing.’ (P01)

‘High risk populations are those clients who are difficult to reach, we have clients like the males, the men who have sex with men. Those are high risk clients because in Eswatini they are discriminated [*against*] and there is stigma attached to being a gay.’ (P11)

‘Factory workers are one of those that don’t have time because factories open at six in the morning and their day ends at eight in the evening sometimes, so they really don’t have time to go to the facility for HIV testing. So, the self-test is really working for us to get those clients who are working at the factories as we target them when they knock off duty in the evenings.’ (P03)

The OPD’s operating hours are weekdays from five in the morning to five in the afternoon, reflecting the need to establish consistent outreach services outside normal working hours to reach more of the high-risk unreachable populations. Outreach activities are typically offered at bus ranks, workplaces, and night mobile clinics to service socially marginalised populations.

#### Theme 2: Views on HIV self-testing

Participants expressed trust in the HIVST’s effectiveness in reaching high-risk, unreachable populations. They felt that eligibility screening for HIVST is reliable to ensure the high-risk populations are not overlooked. However, they raised concerns that the HIVST is not always accessible to all in the targeted high-risk populations. They felt that access to HIVST services is currently compromised because most HIV self-tests were only accessible through a healthcare facility. The HIV counsellors also indicated that ineligible populations were unintentionally reached through HIVST, but that it has an additional advantage of attracting them to preventive services:

‘Self-testing as a strategy for me to reach high risk population. This strategy is very reliable.’ (P02)

‘For now, I think it is reaching some targeted populations but not effectively because you have to access it at certain spaces but not everywhere, you need to access it in health facilities so I think in that way it is not reaching everyone as intended.’ (P13)

Participants further reported that the target population regards HIVST as acceptable in reaching them with tailored HIV testing services. They regard HIVST as convenient. The risks of stigma were not regarded as a concern because the test can be performed in private by the user:

‘This screening approach overcomes the initial stigma of HIV testing by promoting privacy and security. On top of it, it is regarded as convenient, confidential and reassuring.’ (P01)

‘HIVST offers privacy, reduces stigma and discrimination as with the HIVST kit the patient or the client is able to take the test at her or his comfortable space including at home.’ (P08)

The HIV counsellors regarded HIV self-testing as a gateway to other HIV services, including linkages to treatment and prevention services. HIV-positive clients are linked (referred) to initiate antiretroviral therapy, while HIV-negative clients gain access to prevention services, including pre-exposure prophylaxis (PrEP), post-exposure prophylaxis (PEP) and voluntary medical male circumcision (VMMC):

‘When a client has screened positive, I confirm the results using the rapid tests and link client to relevant HIV services, for example HIV treatment. I link clients to prevention services which is PrEP, and condoms.’ (P01)

‘It also creates demand for HIV prevention services because once a person uses the HIV self-test and find that he is not HIV positive, they are now more likely to access HIV preventive services which include, pre-exposure prophylaxis, voluntary medical male circumcision and post-exposure prophylaxis.’ (P04)

Another positive view was that HIVST has relieved some of the HIV counsellors’ workload without compromising the targeted services. The fact that HIVST can be performed at clients’ convenience and in a private space, away from the HIV testing site, was viewed as reducing HIV counsellors’ workload. Reducing workload is of particular benefit in the context of the growing quadruple burden of disease. Participants expressed some relief in performing added tasks because HIVST ensures their main duty of providing HIV testing is not compromised, and no clients are overlooked in accessing HIV testing services:

‘HIV self-test relieves counsellors from the workload since clients are able to test themselves and come for confirmation if a need arises.’ (P01)

‘As I have mentioned before, our facility is one of the high-volume facilities in the country, so HIV self-test help us to reach more, without HIV counsellors getting exhausted.’ (P03)

#### Theme 3: Recommendations for improving HIV self-testing provision as a targeted HIV service

Participants recommended areas for improving HIVST beyond targeting. They also shared the areas that require support to improve targeted HIVST services. The recommendations included client involvement, building HIV counsellors’ HIVST capacity and government support.

Client involvement in shaping HIVST programming was viewed as critical to ensure efficiency. Most participants recommended a feedback mechanism for providers and clients to improve the quality of the HIVST service. Physical and virtual platforms were suggested by participants for education and feedback of the targeted populations. Health education can include campaigns, promotions on social spaces and using social media platforms:

‘… I also recommend that if we can create a platform whereby you can get feedback from the high-risk population groups on how they want us to offer the services and how to pack the HIV self-test so that they can feel free and flexible to use it.’ (P04)

‘I think we should provide reliable contact support lines, to enable clients to feel free to ask questions and make any recommendations regarding the kit or even ask where they can access the kit themselves.’ (P12)

Participants understood that they may not all be at the same level of competency in the provision of HIVST as part of targeted HIV services and expressed the need for frequent refresher trainings, for example, quarterly. Refresher trainings could include site-specific trainings as targeted populations differ from site to site:

‘Refresher trainings on how to provide HIVST is necessary. At the moment we do receive trainings once or twice a year, so I feel like if the trainings can be frequent, maybe every quarter, to review our targeted testing strategy. Also, the refresher trainings will help in ensuring that HIV counsellors are at the same level.’ (P10)

‘I think there is a need for more trainings, you know that the health sector is very dynamic, so we need to have frequent trainings, we have updates on the strategy, on HIV self-test strategy like on how far have we achieved.’ (P11)

Participants indicated the importance of government support through policies, strategies and resource supply because of the dependence thereof for a sustainable HIVST programme. A constant supply of HIVST kits was recommended to enable uninterrupted targeted HIV self-test services. The supply of HIVST kits was a challenge:

‘We know that in the past we had a shortages of HIV self-tests so if government can improve on that, I think it can be much better.’ (P07)

‘I think the stock should be always available, it should not dry out so that we make sure that every time the clients need the self-testing it is available.’ (P08)

Participants suggested a review of the HTS guidelines especially for clients when accessing HIV prevention services such as PrEP. The HIV counsellors recommended having HIV self-tests approved as a stand-alone test for HIV-negative clients. This could build more trust in the test and improve its use among high-risk and hard-to-reach populations. Participants felt that the screening process might have created doubt among the targeted population about HIVST’s importance in their healthcare plan, thus ignore the need to utilise the service:

‘I can recommend that HIVST be approved or recognised as a test to reduce the time used for HIV testing … specially to access prevention services like PrEP.’ (P08)

‘For a client who fear the pricks with rapid testing, you will find that the client eventually does not access any HIV service yet have been on PrEP for 2 years but because is required to go via the rapid testing each time for refills. So, if we can have self-test as enough test for PrEP refills, that I think that will be much easier and then it allows linkages to be much easier.’ (P13)

These findings suggest the importance of the government’s role in improving the efficiency of HIVST services in Eswatini to address HIV.

## Discussion

This study explored the knowledge and attitudes of HIV counsellors in Eswatini regarding HIVST as a targeted strategy for reaching high-risk key populations. The findings reveal that HIV counsellors are well-equipped with knowledge about their roles and responsibilities, enabling them to implement HIVST effectively. In addition, they hold positive views of HIVST, and their insights provide valuable strategies to optimise testing, although challenges remain in fully realising HIVST’s potential as a targeted intervention.

HIV counsellors are key frontline workers in the fight against the HIV epidemic and a lack of knowledge could lead to delayed diagnosis, morbidity, mortality and spread of the disease (Maurya et al. 2022). HIV counsellors in this study demonstrated comprehensive knowledge of HIVST processes, including screening for eligibility, client education, confirming positive results, and linking clients to appropriate treatment and prevention services. These findings are consistent with the WHO guidelines, which highlight the importance of counsellors’ expertise in implementing self-testing as part of HIV services to enhance early diagnosis and linkage to care for vulnerable populations (WHO 2024).

The ability of counsellors to identify high-risk populations, such as men who have sex with men, adolescent girls and factory workers, reflects their understanding of the epidemiological factors driving HIV transmission in Eswatini. This aligns with global evidence suggesting that targeted interventions are critical for addressing disparities in HIV testing uptake among marginalised populations (UNAIDS 2023). The HIV counsellors indicate the need for tailored outreach strategies in the specific areas, for example, to cater for factory workers after working hours.

In addition to knowledge, attitudes influence how people behave (Narang 2020). A key finding in the study is the HIV counsellors’ positive views of HIVST including trust in the effectiveness of HIVST to reach the targeted high-risks populations. By promoting privacy and convenience, HIVST overcomes barriers associated with traditional testing approaches, such as stigma and time constraints. This is particularly relevant in Eswatini, where stigma surrounding key populations remains a major barrier to care. Similar findings have been reported in other sub-Saharan African settings, where self-testing has been shown to improve testing uptake among hard-to-reach groups (Choko et al. 2021).

The study highlighted concerns about limited accessibility to HIV self-tests, as clients are often required to access them through healthcare facilities. This limitation undermines the potential of HIV self-testing to reach its intended populations effectively, especially because HIVST is acceptable to the key at-risk populations (Harichund & Moshabela 2018; Moradi et al. 2022). The WHO recommends expanding self-test availability through community-based models and pharmacies to increase accessibility and coverage (WHO 2019). National guidelines to expand their use as a stand-alone test for prevention services, such as PrEP, could enhance the utility and acceptability of self-testing. This recommendation is in line with what WHO (2023) recommended, which is that countries expand the use of HIVST for initiation, continuation and re-starting PrEP, and that they promote testing through sexual and social networks. If resources allow, a policy that permits HIVST as a free for all, can enhance HIV awareness and early diagnosis.

Participants’ recommendations for improving HIVST services highlight areas for intervention at policy, programmatic, and operational levels. Suggestions to incorporate client feedback mechanisms and expand health education through virtual and physical platforms align with the need for user-centred approaches in HIV service delivery. Evidence suggests that involving clients in designing healthcare services enhances acceptability and utilisation, and reduces stigma (Nyblade et al. 2018).

The study also underscores the importance of frequent refresher training for HIV counsellors. The dynamic nature of healthcare, including updates to HIV testing guidelines and technologies, necessitates continuous capacity-building initiatives. Quarterly refresher training, as recommended by participants, could ensure uniformity in HIV counsellors’ competencies across different settings. Continuous professional development aligns with the WHO’s (2024) aim to ensure quality of HTS to prevent misdiagnosis, which could have serious consequences for public health. Site-specific trainings were mentioned to possibly assist to ensure HIV counsellors are conscious of specific high-risk population profiles at the site and ensure efficient identification and reach of these populations.

Finally, the study addresses the shortage of HIVST kits and the need for government to prioritise an uninterrupted supply of the kits. Previous research has highlighted the importance of consistent supply chains and policy alignment in sustaining HIVST programmes (Aizobu et al. 2023).

There is a need for similar studies to explore knowledge and attitudes of other healthcare providers of HIVST in Eswatini. The relevance of the current HTS curriculum including HIVST provision, should be re-evaluated and adapted to ensure relevance and optimal targeting of high-risk populations. Future studies could explore the impact of task-shifting on HIV counsellors.

### Limitations

Availability of HIV counsellors for the interviews was a challenge as some days’ interviews were delayed because of participants attending to their daily duties. The delay or later hours of the interview might have resulted in distractions, although the researcher mitigated the time constraints challenge by making bookings with participants in advance and reminding them a day before the interview. Participants were made aware of an option to reschedule the interview should a need have arisen.

## Conclusion

This study showed that the knowledge of HIV counsellors regarding HIVST provision was comprehensive and sufficient to ensure targeting, namely to screen clients for HIVST eligibility, educate clients on how to perform HIV self-tests, confirm positive HIV self-test screening results and offer linkages to treatment and HIV prevention services, ensuring service is maximised for the targeted populations. HIV counsellors showed positive attitudes towards HIVST’s effectiveness as a targeted strategy, target population’s acceptability of HIVST, being a gateway to other HIV services as well as reducing HIV counsellors’ workload. Nonetheless, HIV counsellors raised concerns regarding the access to HIVST services, as being dependent on a healthcare worker. The use of HIVST as a screening test requiring another test if one is interested in prevention services such as PrEP and PEP, was further viewed as a barrier to targeting. This denotes that HIV counsellors adequate knowledge and positive attitude can translate into good practices. Advocacy for a revised HTS algorithm and curriculum as well as an uninterrupted supply of HIVST are some of the recommendations to improve targeted HIVST.
